# Distribution Patterns of Benthic Protist Communities Depending on Depth Revealed by Environmental Sequencing—From the Sublittoral to the Deep Sea

**DOI:** 10.3390/microorganisms11071664

**Published:** 2023-06-26

**Authors:** Manon Dünn, Hartmut Arndt

**Affiliations:** Institute of Zoology, Biocenter Cologne, University of Cologne, Zuelpicher Str. 47b, 50674 Cologne, Germany; manon.duenn@uni-koeln.de

**Keywords:** amplicon sequencing, Atlantic Ocean, biogeography, distribution, diversity, unicellular eukaryotes

## Abstract

Protists are key components of the microbial food web in marine pelagic systems because they link algal and bacterial production to higher trophic levels. However, their functioning and bathymetric distribution in benthic deep-sea ecosystems are still only poorly understood. However, biogeographical patterns of communities can be coupled to the functioning of ecosystems and are therefore important to understand ecological and evolutionary processes. In this study, we investigated the diversity and distribution of benthic protist communities from the sublittoral down to the deep seafloor (50–2000 m) around three islands of the Azores in the North Atlantic Ocean. Using amplicon sequencing of the V9 region (18S rDNA) of 21 samples, we found that protist community compositions from different depths were significantly different. Three assemblages were separated along the following depths: 50 m, 150–500 m and 1000–2000 m, which indicate that deep-sea areas surrounding islands might act as isolating barriers for benthic protist species. A limited gene flow between the communities could favor speciation processes, leading to the unique protist communities found at the different investigated islands.

## 1. Introduction

Protists were found to be the most diverse and dominant group of eukaryotes in marine surface and bathypelagic waters [1,2], where they are crucial components of the microbial food web with a wide range of nutritional strategies [3]. Since recent studies using high-throughput sequencing techniques, it is known that protists are also highly diverse in deep-sea ecosystems [4,5]. However, as the deep sea is such a remote and undersampled environment, the origin of this high diversity of protists is still not fully understood [6]. So far, it is known that benthic deep-sea protist communities share only a little part of their diversity with pelagic communities, especially with those from surface waters [4,5]. Biogeographical patterns of protist communities on the deep-sea floor were found at large and even at small spatial scales [7]. Different depth zones of the deep sea, such as the bathyal, abyssal and hadal regions have been shown to possess distinctly different protist communities, and separated ocean basins are inhabited by partly unique protist communities [5,8].

However, little is known so far on the bathymetric distribution of protists from shallow sublittoral, over bathyal depths down to the abyssal deep-sea floor. Although, this knowledge would be especially important to investigate the role of islands and seamounts in the distribution and evolution of benthic protist species. As species might be limited in their distribution by increasing depth due to the changing environmental conditions (decreasing temperature, increasing pressure, absence of light, limited food availability), they may become “trapped” on islands, seamounts or continental shelves. Being isolated from other populations in this way, allopatric speciation processes could possibly be favored. On the other hand, some species might be able to distribute via the deep seafloor using islands and seamounts as kinds of “stepping stones”, enabling a constant gene flow between the populations over large distances [9,10].

A previous study on the Great Meteor Seamount in the North Atlantic Ocean gave the first hints for isolated protist communities on the seamount, which were found to be distinct from communities inhabiting the surrounding deep-sea areas [11]. Studies based on abundance estimations in the Pacific Ocean [12], the Mediterranean Sea [13], the Arabian Sea [14], and the North Atlantic Ocean [15], showed that protist abundances decreased with increasing depth, indicating that several species might be limited in their distribution by the changing conditions with increasing depth. While the theory of oceanic island biogeography is quite well studied for marine meiofauna [9] and reef fish [16,17,18], little is known about its significance for benthic unicellular eukaryotes, which had originally been assumed by some scientists to show no biogeographic patterns [19]; however, this was before genotypes were broadly considered in protist biogeography.

The present study aims to investigate the community composition and bathymetric distribution of heterotrophic protist communities from the sublittoral to the deep sea using the isolated Azores islands (central North Atlantic) as a model region. We analyzed 21 sampling points distributed along depth transects around three Azorean islands, i.e., Flores, Terceira and Santa Maria. We used high-throughput sequencing of the hypervariable V9 region on the 18S rDNA and linked our data to our previous investigations on the same sampling stations based on live observations and cultivation techniques [15].

## 2. Materials and Methods

### 2.1. Sampling

**Figure 1:**
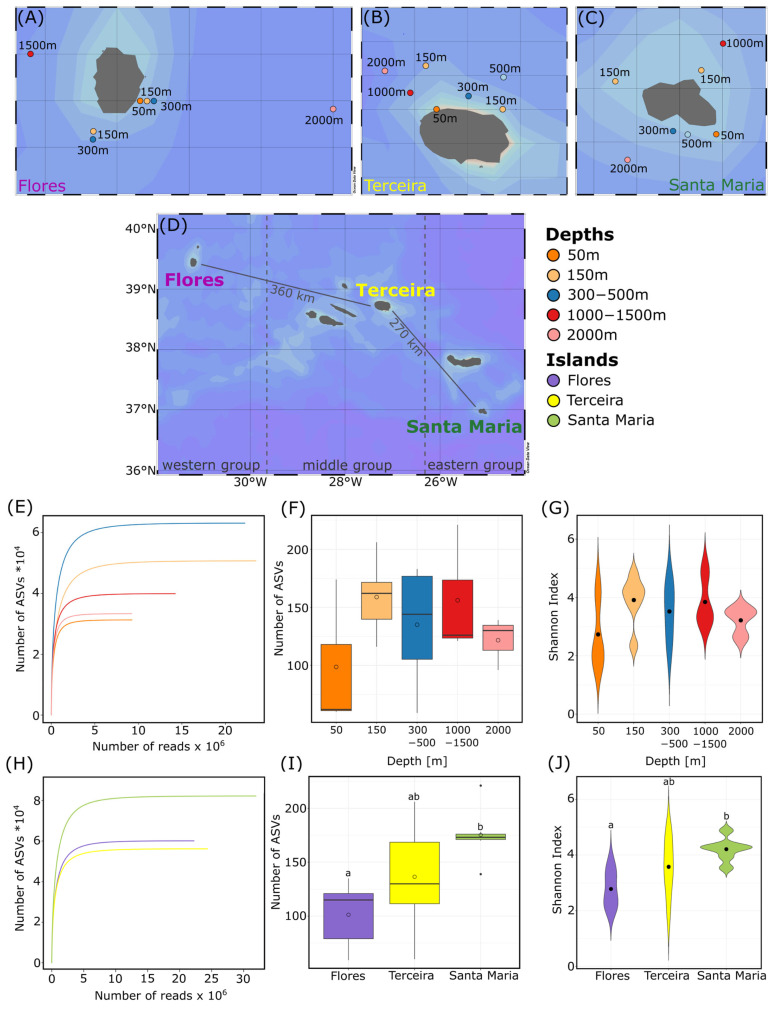
Map of the sampling area around the Azores islands (**A**) Flores, (**B**) Terceira and (**C**) Santa Maria with (**D**) an overview map of the whole archipelago. Maps were created using Ocean Data View (Schlitzer, 2012). (**E**,**H**) Rarefaction curves, (**F**,**I**) number of heterotrophic protist ASVs (amplicon sequence variants) and (**G**,**J**) the Shannon diversity of the investigated depths and islands are shown. In boxplots, circles show mean values, black lines inside the boxes show the median and filled circles show outliers.

Sediment samples were taken at the three Azorean islands Flores, Terceira and Santa Maria during cruise M150 with an R/V Meteor (27 August–3 October 2018; [20]). The sediment was sampled considering different bathymetric strata from sublittoral (50 m, 150 m) to bathyal (300 m, 500 m, 1000 m, 1500 m, 2000 m) depths (Figure 1A–D, Table S1). Three different sampling gears were used due to the different sediment characteristics in different depths, especially considering grain size. The Shipek grab was used for the sampling at the 50 m, 150 m and 300 m depths, while a Boxcorer was used for sampling at 500 m and 1000 m depths. Deeper sediment samples were taken with the help of a multicorer (MUC). Only samples with undisturbed sediment surfaces were used and the surface sediment layer (0–1 cm) was sampled using a sterilized sample spoon. All samples were deep frozen directly after sampling at −80 °C for molecular analysis.

### 2.2. DNA Extraction, PCR Amplification and High-Throughput Sequencing

Prior to DNA extraction, sediment samples were pre-washed with three different washing solutions to improve the success of DNA amplification during PCR by removing potential interfering materials [21,22]. Whole genomic DNA was extracted from 1 g of sediment using the DNeasy Power Lyzer Power Soil^®^ DNA Isolation Kit (Qiagen, Hilden, Germany), using additional heating steps after bead beating [21], and total DNA was quantified using a Quantus Fluorometer (Promega, Walldorf, Germany). Additionally, the hypervariable V9 region on the SSU rDNA was amplified by PCR using the commonly applied eukaryotic primer pair 1389F (5′-TTG TAC ACA CCG CCC-3′) and 1510R (5′-CCT TCY GCA GGT TCA CCT AC-3′) [23]. The PCR mixtures contained 20 ng of total DNA template with a final concentration of 0.35 µM for each primer and the VWR Red Taq DNA Polymerase Master Mix (VWR, Erlangen, Germany). The thermal program consisted of an initial denaturation step at 98 °C for 30 s, 25 cycles at 98 °C for 10 s, 57 °C for 30 s, 72 °C for 30 s and a final elongation step at 72 °C for 10 min. The number of cycles during PCR was set to 25 to avoid chimera formation during the plateau phase of the reaction [24].

Furthermore, the PCR was performed in triplicates to smooth the intra-sample variance. Triplicates were pooled and purified using the FastGene Gel/PCR Extraction Kit (Nippon Genetics, Düren, Germany).

In parallel, we created an artificial community (hereafter called “mock community”), comprising diverse, known protist species, to use as a quality measure of the sequencing process. The DNAs of nine protist cultures (Table S2) were isolated from the HFCC (Heterotrophic Flagellate Collection Cologne) using the Quick g-DNA Miniprep kit (Zymo Research, Freiburg, Germany), which were subsequently amplified by PCR, followed by purification and quantification as described above. Species were selected to cover representatives of the main supergroups. The PCR products of the mock community were then pooled (20 ng of DNA per strain) and added to each Illumina library as a supplementary sample. The same mock community is used in our working group to enable comparative studies [25]. Paired-end NovaSeq sequencing (2 × 150 bp) of amplified fragments was performed by the Cologne Center for Genomics (CCG) of the University of Cologne.

### 2.3. Bioinformatic Processing

Raw reads were demultiplexed and barcoded, and primer sequences were trimmed using cutadapt version 2.8 with the parameters consisting of *no-indels*, *m* = 30 and *e* = 0 for barcodes and *e* = 0.2 for primer sequences [26]. Afterwards, the reads were quality filtered using the *filterAndTrim* command of the dada2 package [27] in R version 4.1.2 using the parameters *maxEE* = 1, *truncQ* = 11 and *truncLen = c*(125, 120) and *maxN* = 0. The error rates were learned using the *errF* and *errR* functions, sequences were dereplicated using the *derepFastq* function and ASVs (amplicon sequence variants) were inferred using the *dada* function. Additionally, paired reads were merged using the *mergePairs* function with a minimum overlap of 12 nucleotides, and chimeric sequences were removed using the *removeBimeraDenovo* function. For the taxonomic assignment, the PR2 reference database (version v4.11.1; [28]) was used, extended by additional sequences of the V9 region of 150 protist strains from the Heterotrophic Flagellate Collection Cologne. ASVs were taxonomically assigned to the reference database using vsearch’s global pairwise alignment function *usearch_global* (version v2.18.0; [29]). Focusing on heterotrophic protists, metazoa, fungi and exclusively phototrophic protists, we removed ASVs, which could not be assigned to a reference sequence (e.g. prokaryotes). Only ASVs with a pairwise identity of >80% to a reference sequence were used for further analysis.

The mock communities were analyzed first, using the bioinformatic processing steps described above. In total, we had five datasets of the mock community because we ran five library preparations and each library included a mock community. Using the mock communities, we used three different filtering procedures: (1) We chose individual, minimum read thresholds per sample for the main dataset, depending on the mock community which ran on the same lane as the respective sample. Therefore, we used the proportion of reads in the ASV with the smallest read number still assigned to one of the nine species included in the mock community (allowing one base mismatch). For example, in all samples which run on the same lane as mock community 1, ASVs with less than 0.09% of the reads in the respective sample were filtered out. For the other mock communities, the minimum read thresholds were similarly high with values of 0.09% (mock 2) and 0.08% (mock 3, mock 4 and mock 5). (2) To analyze if the chosen read thresholds influence observed community patterns, we used a second dataset with a stricter read threshold. Therefore, we chose the minimum proportion of reads in the mock communities necessary to obtain only the nine expected ASVs, resulting in the following thresholds: mock 1 = 0.79%, mock 2 = 0.9%, mock 3 = 0.78%, mock 4 = 0.8% and mock 5 = 0.74% of the reads in the respective sample. (3) By using read thresholds, genotypes only rarely occurring in the environment might be filtered out. Hence, we separately analyzed the ‘low abundant’ ASVs, using a dataset composed of all ASVs filtered out by the read threshold in the main dataset. To avoid ‘noisy’ ASVs, we applied a pairwise identity threshold of >98% to a reference sequence from the database in this ‘low abundant’ ASV dataset.

### 2.4. Statistical Analyses

Statistical analyses were conducted using the software R v4.0.5. Rarefaction curves were constructed to evaluate the sequencing depth, and Shannon indices were computed to analyze the alpha diversity, both using the R package *vegan* [30]. To compare community compositions across different depths, non-metric multidimensional scaling (NMDS) was performed on the dissimilarity matrix based on the Jaccard distance using presence/absence data (functions *vegdist* and *metMDS* in the *vegan* package). Permutational multivariate analysis of variance (PermANOVA) was calculated using the same matrix to test if the community composition of three different depth zones differed significantly (function *adonis2* and *pairwise.adonis2*). To test if the community composition on the three different islands differed significantly, a two-factor nested permANOVA with islands and depths nested within islands was used. Venn diagrams were computed with the R package *VennDiagram* [31] to analyze the number of ASVs shared between multiple or unique to single islands/depths. A number of shared ASVs between different investigated depths and the three islands were visualized using the R package *UpSetR* [32]. Finally, the graphs were plotted using the R package *ggplot2* [33].

## 3. Results and Discussion

### 3.1. Alpha Diversity

NovaSeq sequencing of the 21 sediment samples resulted in 238,217,488 raw, demultiplexed reads, with a mean read number of 11.3 ± 4.9 million reads per sample. During the taxonomic assignment, 55% (43,031,247 reads) of the total assembled and filtered reads (78,465,883 reads) could be assigned to a reference sequence from the database with a pairwise identity larger than 80% (Figure S1), illustrating the high diversity which is uncovered by common reference databases of the V9 region so far. The resolution of metabarcoding studies strongly depends on the chosen marker region in combination with the reference database used. Despite lower numbers of available reference sequences for the V9 region in comparison to, e.g., the V4 region [34], we have chosen this marker region as it covers a high range of diversity, including taxonomic groups highly represented in marine sediments (e.g., some Discicristata, a rare taxa), which are not detected by V4 primers [35]. Moreover, the low average p-identity to reference sequences from the database also shows the high number of, so far, undescribed species present in marine sediments, which we also observed in our previous study based on live observations and cultivation-based methods [15].

About 46% (19,600,535 reads) of these reads were associated with heterotrophic protists after filtering out sequences of metazoans, fungi, streptophytes and exclusively phototrophic taxa. After applying the read threshold chosen with the mock community, filtered reads (13,766,463) in the main dataset clustered into 1370 ASVs. Rarefaction curves of samples from all depths and islands reached saturation, indicating that the sequencing depth was sufficient (Figure 1E,H). The highest average ASV number was reached at 150 m depth with 159 ± 29 ASVs per sample, while the lowest was reached at 50 m depth with 99 ± 53 ASVs (Figure 1F). For reads, the highest average number was found in samples from 50 m depth with 982,639 ± 565,261 reads and the lowest at 150 m depth with an average of 398,356 ± 176,982 reads (Figure S2). Additionally, the Shannon diversity index, a measure of alpha diversity, ranged from 2.7 at 50 m depth to 3.9 at 150 m depth with a mean value of 3.5 (Figure 1G).

Concerning the different islands, the highest ASV number was detected in samples from Santa Maria, with an average of 175 ± 22 ASVs per sample, while the lowest was found at Flores, with an average of 101 ± 28 ASVs per sample (Figure 1I). The highest number of reads was found in samples from Terceira, with an average of 788,257 ± 493,904 reads and the lowest in samples from Santa Maria with 512,542 ± 102,126 reads (Figure S2). The Shannon diversity ranged from 2.8 at Flores to 4.2 at Santa Maria (Figure 1J).

### 3.2. Taxonomic Composition of Communities in Different Depths

**Figure 2:**
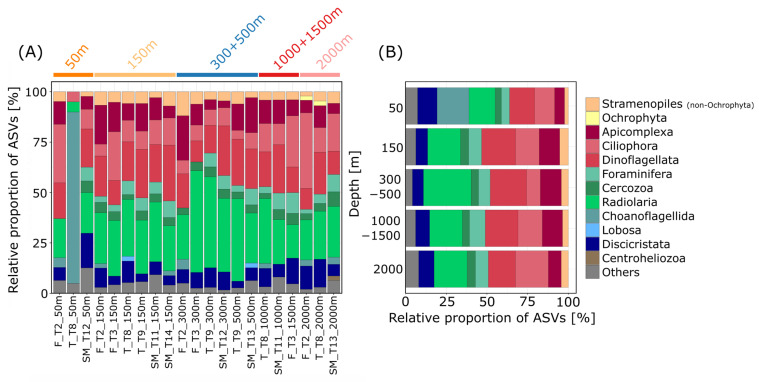
Community composition of benthic heterotrophic protists at different depths. Relative proportion of ASVs (amplicon sequence variants) assigned to major taxonomic groups (corresponding to division level in the PR2 database) within (**A**) all 21 stations and (**B**) for the different investigated depths pooled. Taxonomic groups with relative proportions of <2% of the total ASVs are cumulated as “Others”.

ASVs were dominated in most stations by Radiolaria and Dinoflagellata with relative proportions between 5% (Terceira, transect T8, 50 m depth) and 50% (Flores, transect T3, 300 m depth) and 10% (Flores, transect T2, 2000 m depth) and 28% (Santa Maria, transect T14, 150 m depth), respectively (Figure 2A). The radiolarian group with the largest number of ASVs was the Spumellaria (Polycystinea) with 87 ASVs of which 57 ASVs belonged to the Spumellarida Group I. Other radiolarian taxa with high read abundances were associated with *Sphaerozoum* and *Collozoum* (Collodaria), which were found to have especially high read abundances in samples from 300 to 500 m depths. Radiolaria are, up to now, mainly reported from plankton samples; therefore, ASVs associated with this group could derive from specimens from the water column, which have sunken down to the ocean floor. However, little is known so far on the ecology of radiolarians, as no radiolarian species exists in cultures yet [36].

Most of the ASVs associated with Dinoflagellata (303 ASVs) belonged either to the group of Dinophyceae (54%) or to Syndiniales (44%), which is a poorly studied group so far exclusively consisting of marine parasitic taxa [37,38,39]. Specifically, especially high read abundances were reached by ASVs assigned to the MALV II Clade 7 (Syndiniales). There are only a few species within the group of Syndiniales described so far. They are mostly known from environmental sequencing studies, where they were found to be dominant representatives in surface waters and the deep sea [1,5,38,39]. Most ASVs belonging to the Dinophyceae could not be assigned to any lower taxonomic level, underlining the lack of available sequence data for this group and the unresolved relationships within the so-called core-dinoflagellates [40,41]. We also found a high diversity of ASVs associated with Ciliophora (175 ASVs), which contributed an average proportion of 13% of ASVs per station, with Oligohymenophorea (55 ASVs) and Spirotrichea (51 ASVs) as the most diverse groups. Especially abundant concerning relative read abundances were ciliate ASVs assigned to Oligohymenophorea and Colpodea, both less abundant in intermediate depths (300–500 m). The highest relative proportion of Ciliophora ASVs was found at 2000 m with a value of 20% (Figure 2B), supporting the notion that ciliates are an important component of deep-sea micro-eukaryotic communities and that there is a specific deep-sea ciliate fauna which does not occur in shallow waters [42].

Moreover, Discicristata comprised 7 to 12% of ASVs with high proportions of ASVs (9%, Figure 2B) and reads (average of 12%) at the 2000 m depth. Most of the Discicristata ASVs belonged to the group of Diplonemea (84%). Diplonemids are poorly studied and were, for a long time, considered rare, but more recently they were found to be a dominant group in marine environmental sequencing studies, especially from the deep-sea realm as well as from the photic zone [5,43,44,45,46]. Due to the high numbers of 18S genotypes found in environmental studies, diplonemids were proposed to have diverse ecological functions [46], with roles in predation and/or parasitism on large protists and small metazoans [44,47,48].

Of lower abundance was the group of Stramenopiles, contributing only about 2 to 5% to the total number of ASVs at depths from 50 to 2000 m (Figure 2B). An exception to this observation was ASVs assigned to the genus *Cafeteria* (Bicoecea), which had high read abundances at all depths. Species belonging to the genus *Cafeteria* are known to be highly common and ubiquitously distributed in the world’s oceans and adapted to various habitat types, including the deep sea [49,50]. They attach with their posterior flagella to any surface. Loricated choanoflagellates belonging to the family of Acanthoecidae had high read abundances and a high ASV richness in samples from 50 m depth (Figure 2A and Figure S3). There are only a few species of this family described yet with only a few V9 sequences available in common reference databases, challenging a distinct identification [51,52]. However, Acanthoecidae are usually found associated with substratum as bacterial biofilm or detritus rather than free floating [51].

However, read abundances need to be interpreted cautiously, as some taxonomic groups (e.g., many ciliates, dinoflagellates and radiolarians) have much higher copy numbers of the 18S rDNA gene than others [53,54,55,56,57] and the gene copy number can vary even within taxa (e.g., in foraminiferans [58]). Species-forming colonies consisting of hundreds or even thousands of cells (such as radiolarians of the group Collodaria) can have large effects on read abundances. Moreover, marine sediments, especially in the deep sea, act as “DNA reservoirs” not only containing the DNA of the inhabiting species but also from the water column above [59,60]. Pelagic species attached to particles or encysted specimens sink down to the ocean floor. Therefore, some ASVs are likely derived from planktonic species (e.g., Radiolaria and Foraminifera). Some taxonomic groups harbor both benthic and pelagic species (e.g., Ciliophora, Dinoflagellata and Foraminifera) and the reference databases are not yet sufficient to discriminate between them. Nevertheless, it was found that spatial patterns in deep-sea sediments are not significantly influenced by “dead” DNA from the water column [61].

### 3.3. Depth-Dependent Patterns of Protist Communities

**Figure 3:**
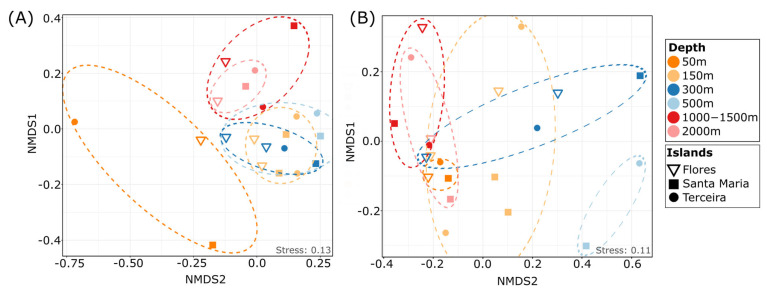
Dissimilarity of heterotrophic protist communities in different depths. NMDS plot based on the Jaccard distance comparing protist communities of the different investigated depths in the main dataset (**A**) and in the dataset with strict filtering criteria (**B**).

The taxonomic composition of communities did not show clear differences between different depths at the taxonomic level (Figure 2A). However, NMDS analysis revealed separated clusters of protist communities at three different depth zones: 50 m (sublittoral), 150–500 m (sublittoral to upper bathyal) and 1000–2000 m (lower bathyal), with significant differences in community composition (permANOVA, *p* < 0.01, Figure 3A), indicating patterns of specific benthic protist communities in relation to depth.

**Figure 4:**
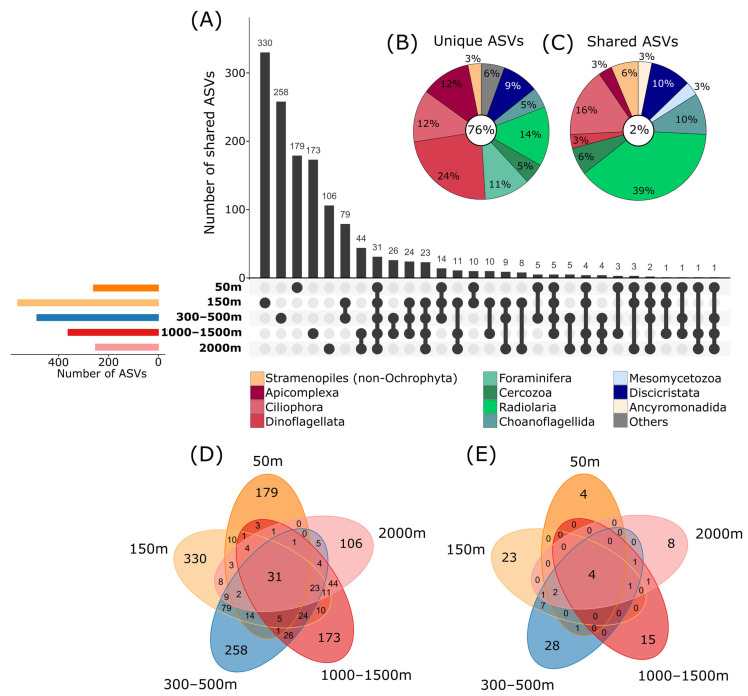
Shared and unique ASVs (amplicon sequence variants) between different depths. (**A**) Upset plot showing in the top bar chart the number of ASVs shared between multiple depths or unique to single depths. Linked dots under the bar chart indicate which depths are compared in the bars above (e.g., 31 ASVs are shared between all 5 depths, indicated by the five dark dots which are linked by a black line). Colored bars on the left show the total number of ASVs per depth. (**B**) Relative proportions of ASVs unique to one depth and (**C**) shared between all depths assigned to major taxonomic groups (corresponding to division level in the PR2 database). Taxonomic groups with relative proportions of <2% of the total unique/shared ASVs are cumulated as “Others”. Percentages in the white circles show the amount of unique and shared ASVs as a percentage of the total protist ASV number. (**D**) Venn diagram showing the number of ASVs unique and shared between depths in the main dataset and (**E**) in the dataset with strict filtering criteria.

The highest number of ASVs unique to one depth was reached in the 150 m depth level, with 24% of the total ASVs, followed by the 300–500 m (19% of ASVs) and 50 m (13% of ASVs) depths, indicating specific protist communities in lower depths, which might be limited in their distribution to deeper areas (Figure 4A,D). About 13% and 8% of ASVs were unique to 1000–1500 m and 2000 m depths, respectively, indicating the presence of species in these depths which are specialized to the prevailing conditions in deeper zones. In the current study, only about 2% of ASVs were shared between all investigated depths, showing that probably only a small part of the protistan composition is widely distributed and most species are specialized to certain depths (Figure 4C), indicating patterns of protist community structure, even at a smaller spatial scale [7]. The significant separation of sublittoral/upper bathyal communities (50–500 m depth) and lower bathyal (1000–2000 m) communities was also evident in the dataset filtered by the rigid filtering criteria (as described in the Section 2; PermANOVA *p* < 0.05; Figure 3B and Figure 4E). Similar patterns were observed in our former study using live observations and cultivation techniques, showing that benthic protist communities from sublittoral and shallow bathyal depths were significantly different in their taxonomic composition compared to communities from deeper areas [15].

Taxonomic groups with the highest proportions of ASVs unique to single depths in the main dataset were Dinoflagellata and certain Ciliophora, Apicomplexa, Discicristata, Radiolaria and Foraminifera, indicating that these groups are strongly influenced by the changing environmental conditions with increasing depth (Figure 4B). For metazoans, it is known that there are clear depth-related distribution patterns. A typical example is those discovered for isopods [62]. Potential metazoan parasites, such as Apicomplexa or Diplonemida, might be influenced by the vertical distribution of their hosts. Radiolaria accounted for 39% of the ASVs shared between all depths. As mentioned above, Radiolaria are predominantly inhabitants of the pelagic zone. Therefore, it is possible that these ASVs originate from planktonic species sunken down from the water column to the sediment, leaving a signal in all investigated depths and reducing differences between sites (Figure 4C).

### 3.4. Genotype Distribution of Cultivated Flagellates

The samples analyzed in this study were also used for a cultivation-based approach [15], resulting in several monoclonal cultures belonging mainly to Cafeteriaceae [50] and Percolomonadida [63]. We used these groups as model groups to check whether we can find V9 sequences belonging to our cultivated strains in the metabarcoding dataset. Only ASVs with a pairwise identity of 100% to a cultivated strain were considered. Sequences identical to the V9 region of *Cafeteria burkhardae* were found with high read abundances in all samples of our metabarcoding dataset. Regarding the percolomonads, three of the four cultivated strains could be found in the dataset. V9 sequences with a 100% similarity to *Percolomonas adaptabilis*, *Lula levis* and *Nonamonas santamariensis* were found in samples from all three islands and from depths between 50 and 2000 m, while sequences identical to the V9 region of *Nonamonas montiensis* were not found.

The high proportion of rediscovered sequences of cultivated strains supports the reliability of the dataset analyzed in this study. Both *Cafeteria burkhardae* and *Percolomonas adaptabilis* were previously described to be widely distributed in marine habitats all over the world [49,63]. Our findings support their cosmopolitan distribution and show that they are also widely distributed in benthic habitats around the Azores islands at various depths. The species *Lula levis* and *Nonamonas santamariensis* were not found in two major metabarcoding datasets in our previous study [63], leading to the assumption that they are restricted in their distribution. However, the current dataset suggests that they are as well widely distributed around the Azores islands in a wide range of depths.

### 3.5. Patterns of Protist Communities at Different Islands

**Figure 5:**
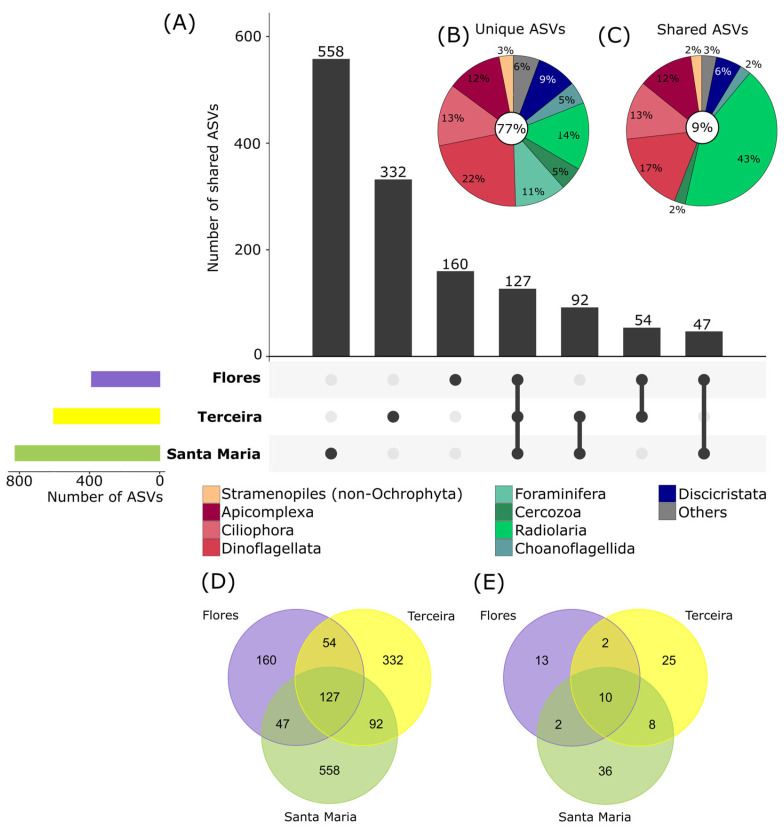
Shared and unique ASVs (amplicon sequence variants) between different investigated islands. (**A**) Upset plot showing in the top bar chart the number of ASVs shared between multiple islands or unique to single islands. Linked dots under the bar chart indicate which islands are compared in the bars above (e.g., 127 ASVs are shared between all 3 islands, indicated by the three dark dots which are linked by a black line). Colored bars on the left show the total number of ASVs per island. (**B**) Relative proportions of ASVs unique to one island and (**C**) shared between all islands assigned to major taxonomic groups (corresponding to division level in the PR2 database). Taxonomic groups with relative proportions of <2% of the total unique/shared ASVs are cumulated as “Others”. Percentages in the white circles show the amount of unique and shared ASVs as a percentage of the total protist ASV number. (**D**) Venn diagram showing the number of ASVs unique and shared between islands in the main dataset and (**E**) in the dataset with strict filtering criteria.

Protist community composition also differed significantly between the three investigated islands (PermANOVA, *p* < 0.01), as well as for depth nested by islands (*p* < 0.01). High numbers of ASVs were unique to single islands (77%, Figure 5A,B,D), and only a low amount of ASVs was shared between all three (9%, Figure 5C). The oldest island, Santa Maria (8.12 mya, [64]), showed the highest diversity with 824 ASVs of which 558 ASVs only occurred at this site (Figure 5A,D). The island Terceira, which emerged 3.52 mya [64], had a lower unique diversity with 332 ASVs, while the youngest island Flores (2.16 mya, [64]) had the lowest unique diversity with 160 ASVs, fitting to the concept of island biogeography [10,65], which states that older islands harbor a higher species richness. The same pattern is also visible using the strict filtering criteria (Figure 5E). However, additional data on more islands and depths are needed to prove this assumption due to the potential variability of benthic protist communities.

In general, we used strict filtering criteria using a mock community to choose individual read thresholds per library preparation, which is an objective measure to get rid of “noisy” ASVs (e.g., [66]). However, we potentially also reduced the diversity present by filtering out low abundant ASVs. A separate analysis of this “low abundant” part of the community (see Section 2) showed similar patterns to the main dataset (Figure S4), with high numbers of ASVs unique to singe islands, but with a higher proportion of ASVs shared between all three islands.

## 4. Conclusions

Our results on the diversity, composition and distribution of benthic protists from the sublittoral to the deep sea obtained by metabarcoding of the V9 region of the 18S rDNA revealed depth-related patterns of protist communities. Higher numbers of unique ASVs were detected, either in the sublittoral (50–150 m) or upper bathyal (300–500 m) regions in comparison to the deepest depths (lower bathyal; 1000–2000 m), indicating that many species seem to be limited in their distribution and restricted to certain depths. ASVs were dominated at all depths by Dinoflagellata, Ciliophora and Radiolaria. Dinoflagellata and Ciliophora seemed to also be strongly influenced by the changing environmental conditions with increasing depth, while high numbers of Radiolaria ASVs probably sedimented from the water column, and this effect might be magnified by colony-forming species. Differences in the composition of benthic protist communities at different islands were revealed by high amounts of ASVs unique to single islands and low numbers of ASVs shared between them. This observation supports the hypothesis that many protist species are limited in their distribution and restricted to lower depths due to missing adaptations to deep-sea conditions. A biogeographical isolation of species on islands, separated by surrounding deep-sea areas, could support speciation processes, leading to unique protist communities.

## Data Availability

The data analyzed in this study are deposited in the Sequence Read Archive SRA under the BioProject ID: PRJNA985410, Sample IDs: SAMN35790901-SAMN35790921.

## Supplementary Materials

The following are available online at https://www.mdpi.com/article/10.3390/microorganisms11071664/s1. Figure S1: Relative sequence similarity of ASVs to sequences from the reference database, Figure S2: Number of reads at the different investigated depths and islands, Figure S3: Relative proportion of reads assigned to major taxonomic groups, Figure S4: Analysis of low abundant ASVs, Table S1: Sampled stations, Table S2: Mock community.
