# Supplementary material for: Distribution Patterns of Benthic Protist Communities Depending on Depth Revealed by Environmental Sequencing—From the Sublittoral to the Deep Sea
Source: Microorganisms. 2023 Jun 26;11(7):1664. doi: 10.3390/microorganisms11071664 (PMC10385078; doi:10.3390/microorganisms11071664)
Supplement: Supplementary file 1 [file microorganisms-11-01664-s001.zip › microorganisms-2437932-supplementary.pdf]

Supplementary Figures and Tables to

Distribution Patterns of Benthic Protist Communities Depending on Depth Revealed by Environmental Sequencing—From the Sublittoral to the Deep Sea

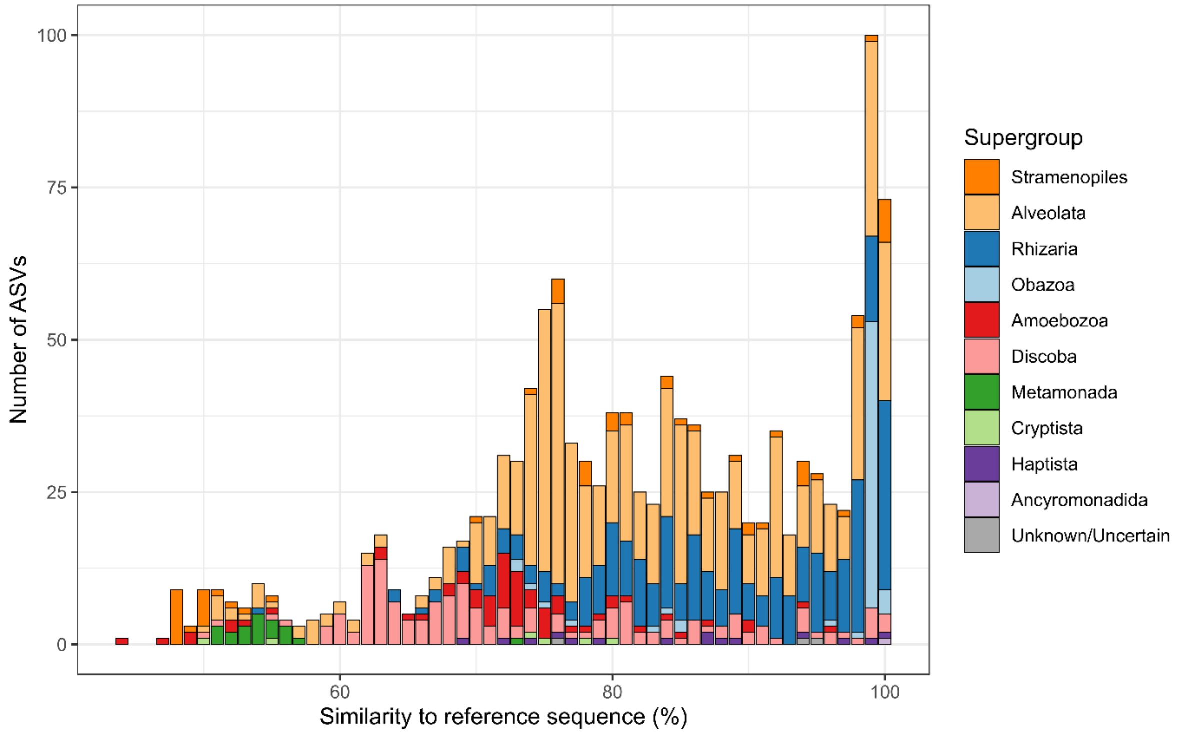

Figure S1. Relative sequence similarity of ASVs to sequences from the reference database. ASVs were assigned to major taxonomic groups.

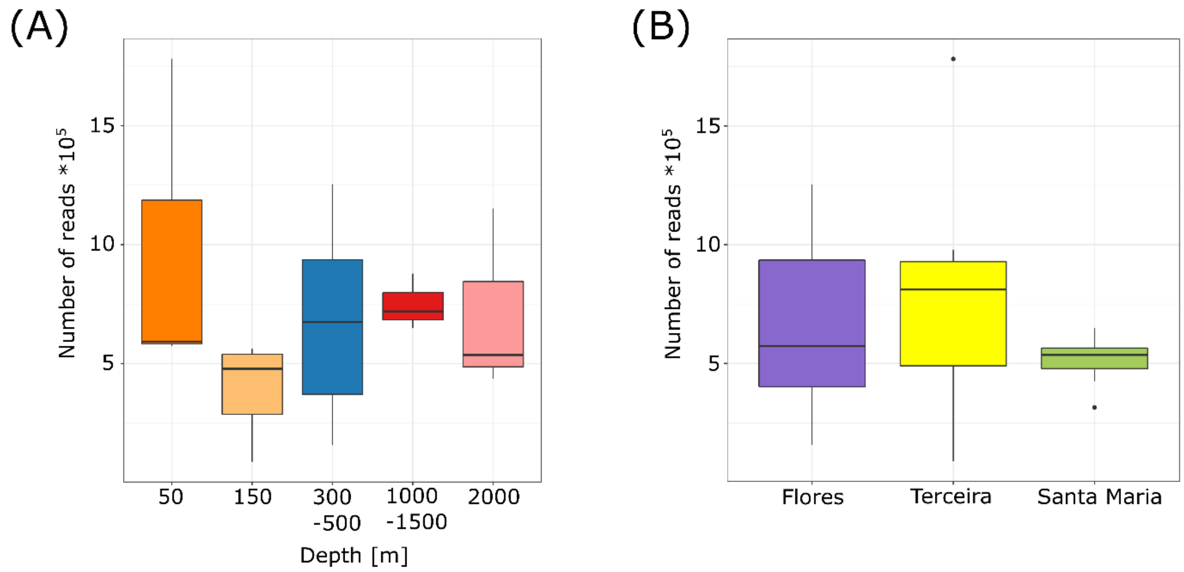

Figure S2. Number of reads at the different investigated (A) depths and (B) islands.

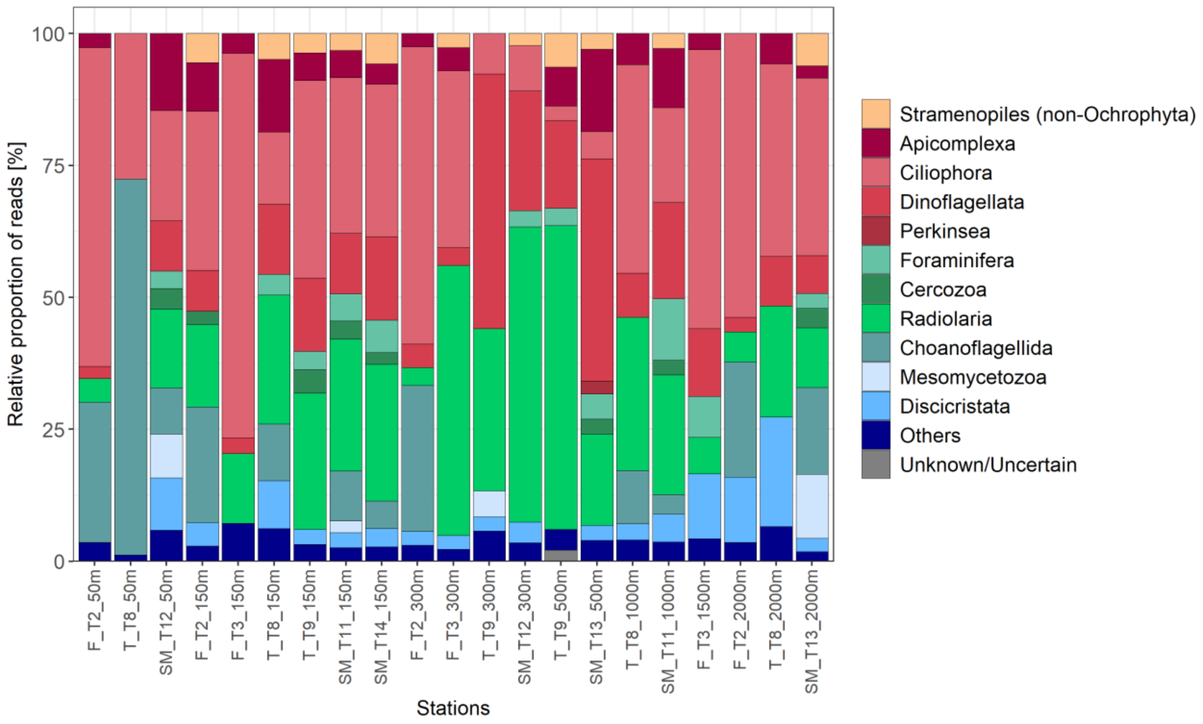

Figure S3. Relative proportion of reads assigned to major taxonomic groups (corresponding to division level in the PR² database) within all 21 stations. Taxonomic groups with relative proportions of < 2% of the total ASVs are cumulated as “Others”.

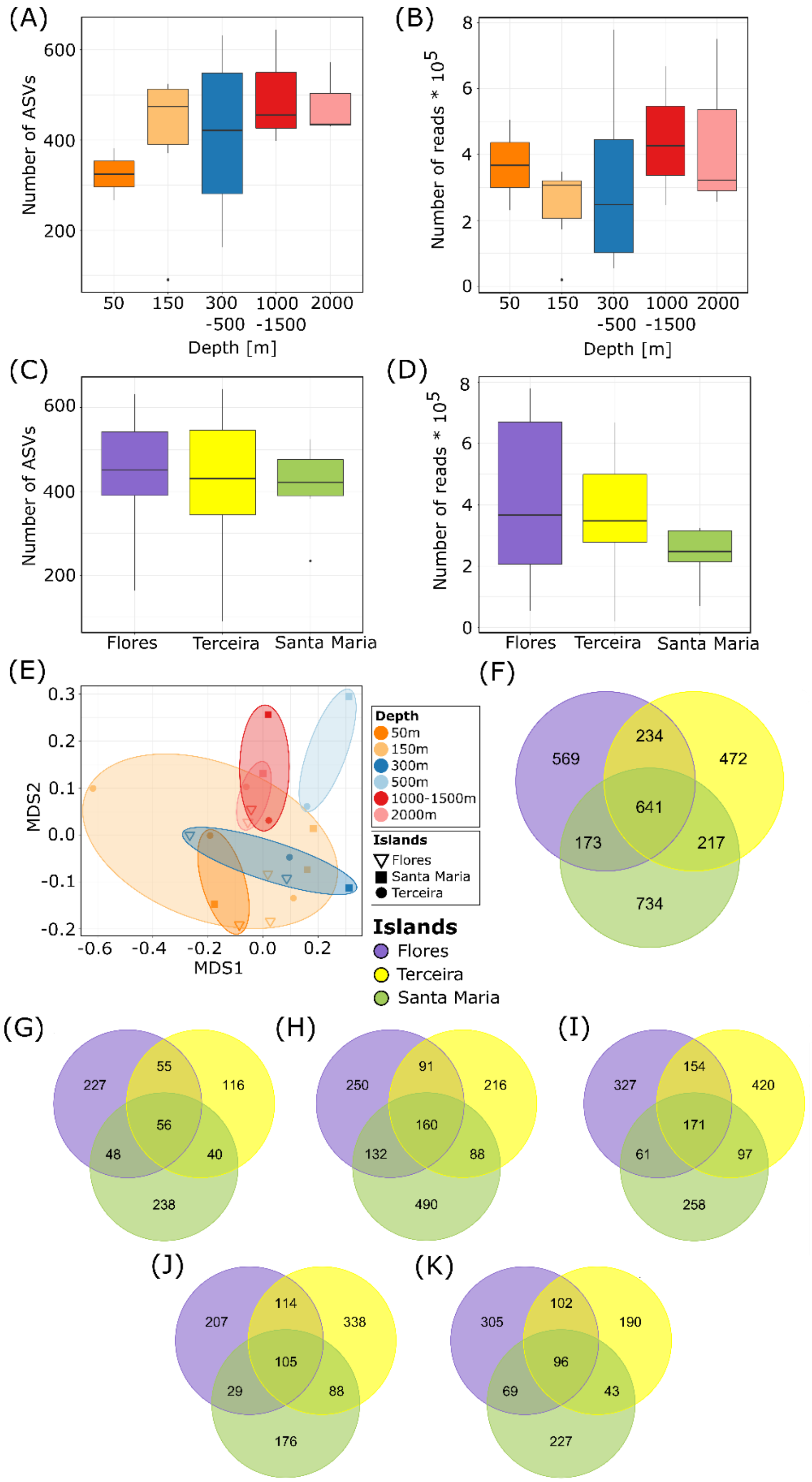

Figure S4. Analysis of low abundant ASVs which are filtered out by the read threshold chosen with the mock community (see Material and methods). Number of ASVs and reads at different investigated (A, B) depths and (C, D) islands. (C) NMDS plot based on the Jaccard distance comparing protist communities of the different investigated depths in the low abundant ASVs dataset (Stress:0.15). Venn diagrams showing unique and shared ASVs between different investigated islands for all investigated depths (F), only for 50m depth (G), 150m depth (H), 300-500m (I), 1000-1500m (J), 2000m (K).

Table S1. Sampled stations. Shown are gear numbers, name of the island around which the sample was taken, transect number, depth category, coordinates of sampling point, the exact depth at sampling and the gear type (SG = Shipek grab, MUC = Multicorer, BC = Boxcorer).

Gear nr.	Island	Transect	Depth Category [m]	Longitude	Latitude	Depth [m]	Gear Type
#59	Flores	T2	50	31° 08,367' W	39° 24,574' N	57	SG
#62	Flores	T2	150	31° 07,303' W	39° 24,347' N	119	SG
#45	Flores	T2	300	31° 06,833' W	39° 24,251' N	323	SG
#74	Flores	T2	2000	30° 36,731' W	39° 23,321' N	2076	MUC
#106	Flores	T3	150	31° 16,054' W	39° 20,071' N	153	SG
#85	Flores	T3	300	31° 16,388' W	39° 19,700' N	257	SG
#93	Flores	T3	1500	31° 18,512' W	39° 15,906' N	1518	BC
#242	Terceira	T8	50	27° 19,294' W	38° 48,153' N	48	SG
#253	Terceira	T8	150	27° 20,092' W	38° 48,646' N	151	SG
#273	Terceira	T8	1000	27° 25,071' W	38° 51,588' N	992	BC
#279	Terceira	T8	2000	27° 31,342' W	38° 55,294' N	2019	MUC
#300	Terceira	T9	150	27° 04,256' W	38° 48,134' N	153	SG
#288	Terceira	T9	300	27° 03,526' W	38° 48,146' N	281	SG
#310	Terceira	T9	500	27° 03,066' W	38° 48,350' N	482	SG
#343	Santa Maria	T11	150	25° 03,411' W	37° 02,810' N	153	SG
#351	Santa Maria	T11	1000	25° 00,610' W	37° 05,199' N	1338	MUC
#371	Santa Maria	T12	50	25° 01,313' W	36° 55,045' N	50	SG
#362	Santa Maria	T12	300	25° 01,359' W	36° 54,077' N	321	SG
#420	Santa Maria	T13	500	25° 05,259' W	36° 55,171' N	505	BC
#430	Santa Maria	T13	2000	25° 10,202' W	36° 51,064' N	2005	BC
#454	Santa Maria	T14	150	25° 09,264' W	37° 03,298' N	153	SG

Table S2. Mock community. Strains of the HFCC culture collection used as mock communities.

HFCC No.	GenBank Accession Number	Species	Supergroup
203	MN315604	*Cafeteria burkhardae*	Stramenopila
176	MT355122	*Massisteria* sp.	Rhizaria
178	MT355150	*Ministeria vibrans*	Obazoa
171	MT355133	Rynchomonadidae sp.	Discoba
828	MT355124	*Neobodo* sp.	Discoba
744	MT081566	*Aristerostoma* sp.	Alveolata
175	MT355148	*Fabomonas tropica*	Ancyromonadida
766	MT355146	*Protocruzia* sp.	Alveolata
768	MT355117	Bicosoecida sp.	Stramenopila
